# Telomerase Inhibitors from Natural Products and Their Anticancer Potential

**DOI:** 10.3390/ijms19010013

**Published:** 2017-12-21

**Authors:** Kumar Ganesan, Baojun Xu

**Affiliations:** Food Science and Technology Program, Beijing Normal University–Hong Kong Baptist University United International College, Zhuhai 519087, China; kumarganesan@uic.edu.hk

**Keywords:** telomere, telomerase inhibitors, natural products, anticancer

## Abstract

Telomeres and telomerase are nowadays exploring traits on targets for anticancer therapy. Telomerase is a unique reverse transcriptase enzyme, considered as a primary factor in almost all cancer cells, which is mainly responsible to regulate the telomere length. Hence, telomerase ensures the indefinite cell proliferation during malignancy—a hallmark of cancer—and this distinctive feature has provided telomerase as the preferred target for drug development in cancer therapy. Deactivation of telomerase and telomere destabilization by natural products provides an opening to succeed new targets for cancer therapy. This review aims to provide a fundamental knowledge for research on telomere, working regulation of telomerase and its various binding proteins to inhibit the telomere/telomerase complex. In addition, the review summarizes the inhibitors of the enzyme catalytic subunit and RNA component, natural products that target telomeres, and suppression of transcriptional and post-transcriptional levels. This extensive understanding of telomerase biology will provide indispensable information for enhancing the efficiency of rational anti-cancer drug design.

## 1. Introduction

Telomerase was initially investigated in the transformed cervical carcinoma (HeLa) cell line in 1989 [1]. In eukaryotes, terminal bases of a linear DNA molecule cannot replicate by normal DNA polymerases and primases. Due to the lacking mechanisms, in each round of DNA replication, chromosomes will shorten with the abolishing of terminal RNA primers [2,3]. Telomerase is a ribonucleic reverse transcriptase enzyme, which reimburses for the loss of those telomeric sequences by connecting tandem repeats at the 3′ end of chromosomes, which produce the telomeres. This enzyme adds nucleotide repeats to telomeres by using RNA template providing karyotype stability and compensating for the loss of DNA replication [4]. However, in normal human somatic cells, this enzyme shows little or no telomerase activity. The telomeric DNA is eventually shortened with each cell division [5]. Telomeres are regions of non-coding DNA constrained at the end of each chromosome whose length indicates life expectancy and overall health status. Based on the structural and functional aspects, telomeres are unique and district from other chromosomal DNA. Telomeres are sequenced by short pattern tandem repetitions of hexanucleotide (TTAGGG) in all eukaryotic organisms. They are essential components that stabilize the ends of eukaryotic chromosome and avoid the loss of genetic information [6]. Telomeres normally defend the chromosome from DNA damage and exonucleolytic degradation, and prevent aberrant recombination and chromosome-to-chromosome fusion. The average lengths of telomeres vary from species to species. Telomeres normally help to control the proliferative capacity of normal somatic cells [7]. 

Eukaryotic telomerase contains a catalytic protein subunit known as telomerase reverse transcriptase component (hTERT), which is conserved by reverse transcriptase (RT) enzymes. In addition, telomerase contains an integral RNA component (hTR), which is essential for the synthesis of the telomeric repeats [8]. Telomerase is predominantly expressed in human tumors and tumor-derived cell lines, about 85–90%. However, in the normal stem cell, this enzyme activity is proportionally low [9]. The function of telomerase is mainly involved in telomere capping and responding to DNA-damage [10]. Telomere length is maintained in human tumors by many factors other than telomerase activity. Normally, the level of telomerase activity is high in tumor cells; in addition, telomere length is further regulated by recombinant factors called as alternative lengthening of telomere (ALT) [7]. The absence of telomerase activity in ALT causes chromatin/methylation remodeling of the catalytic proteins, hTERT and hTR [11]. Based on the catalytic activities and recombination factors, the telomere is highly heterogeneous in length in cancer cells. Based on the telomere maintenance, unlimited cell proliferation occurs in cancer [12]. Telomerase could be a reliable marker and potential target for some important cancers; however, it does not play a role in all cancers or immortal cell growth inhibitors [13]. Only 15% of cancers enable its telomere by ALT. Besides that, the development of telomerase inhibitors as anti-cancer agents is reasonable and feasible. Hence, most telomere-related antitumor strategies target telomere maintenance through the telomerase-dependent mechanism. Numerous telomerase inhibitors have been produced and inhibit the catalytic activity of enzyme through the targeting of its catalytic components or RNA. Telomerase inhibitors are generally diverse compounds, including natural as well as synthetic products and modified oligonucleotides [7]. Telomere binding agents such as quadruplex ligands (G4) can play a role in both telomerase positive and ALT cells. However, based on the available literature, there is no inhibitor specifically for the ALT mechanism.

**Figure 1:**
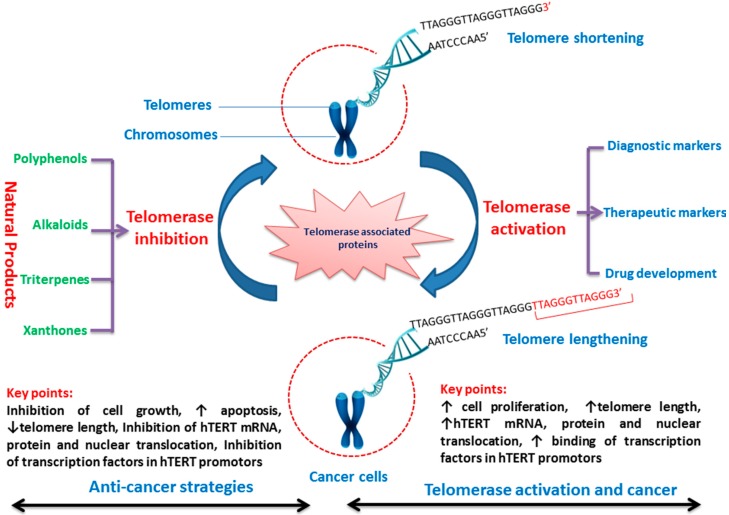
Telomerase-related anticancer strategies by natural products. ↑: increase; ↓: decrease; hTERT: human telomerase reverse transcriptase.

Telomerase is generally observed in most cancer cells and is critical for cancer cell development [14]. Hence, the deactivation or inhibition of telomerase is essential in the cancer-suppressive mechanism. The deactivation of telomerase and destabilization of telomere by natural/synthetic products provides extensive opportunity to succeed new targets for cancer therapy. Nowadays, several synthetic compounds are commercially used for chemotherapy of cancer. However, most have many side-effects or complications in the cancer patients. Hence, it is very important to explore the beneficial effects of natural products such as medicinal plants on the various cancer cells and potential anti-cancer therapeutic effects [15]. Moreover, natural products are normally taken in the human diet as the traditional medicine that are edible, safe to consume and have higher acceptability among the individuals [16]. Besides that, natural products reduce/inhibit the telomerase activity that can be utilized as functional food by the cancer individual for healing or treatment. Thus, this review puts forward the use of natural products that inhibit the telomerase as a phytomedicine in cancer prevention, which can be noted as a direction for future research on targets for cancer therapy (Figure 1). Furthermore, this review aims to provide fundamental knowledge for research on the telomerase structure, functions, working regulation of telomerase and its various binding proteins to inhibit the telomere/telomerase complex. In addition, the review summarizes the inhibitors of the enzyme catalytic subunit and RNA component, natural products that target telomeres and suppression of transcriptional and post-transcriptional levels.

Telomeres are normally located at a terminal of the chromosomes of all organisms, comprising DNA. The repetitive sequences of telomeric DNA rich in guanine with a single-stranded 3′ end, which folds onto the double-stranded telomere and, eventually, becomes a t-loop structure. This t-loop structure causes cap formation at the chromosome ends, which protects from degradation, recombination, and end-to-end fusion. Telomere is generally able to maintain a certain length of the strand through telomerase enzyme and regulatory proteins. Several telomeric proteins, telomerase components and telomere repair proteins are required to maintain certain tasks by binding with single/double-stranded telomeric DNA. Significant double-strand telomere DNA-binding proteins include telomeric repeat binding factor 1 (TRF1) and telomeric repeat binding factor 2 (TRF2), which are responsible for formation of t-loop and telomere complex. Furthermore, telomere is conserved through the complex formed by these regulatory special proteins [17,18,19,20]. The other telomere proteins that compose this complex and their duties are briefly summarized in Table 1. 

## 2. Expression of Telomerase in Cancer Cells

The levels of telomerase activity in the early and late stage of cancer might be used to determine the diagnosis of various human cancers (Table 2). Based on the levels of this enzyme, tumor behavior such as differentiation and metastasis of cells could be determined. The higher expressions of telomerase activity in cells have been associated with poor differentiation and higher mortality incidence in patients with adenocarcinoma and small cell cancer of the lung [49,50]. Similarly, high expression of telomerase is found in patients with breast cancer (86%) [51], colorectal cancers (80–90%) [52] and gastrointestinal cancer (70%) [53]. The expression of telomerase in most cancer cells is directly proportional to the expression of hTERT mRNA [54,55]. Nevertheless, some cancer cells do not express telomerase. For incidence in breast cancer cells, there is a higher expression of hTERT mRNA and protein without active telomerase [56]. In another study, a high expression of hTERT mRNA with telomerase activity was demonstrated with advanced stages of colonic adenocarcinoma and endometrial cancer [57]. These findings relate to a high degree of malignancy that could be used as a diagnostic marker to detect cancer with high recurrence rate [58]. 

Telomerase activity in cancer cells is normally inhibited by various natural products, and this inhibition has been connected with the decrease of cell viability [74]. The therapeutic effect of natural products on various cancers decreases telomerase activity by down-regulation of the hTERT mRNA expression, apoptosis induction and induce senescence via the DNA damage response. In addition, these natural products activate p53 expression that inhibits cell cycle, migration and metastatic ability [70,72]. Therapeutic implications of telomerase in various human cancers by natural products on various human cancers are listed in Table 2.

## 3. Telomerase Inhibitors from Natural Products

Telomerase inhibitors, commonly derived from natural plant materials, include secondary metabolites such as polyphenols, alkaloids, terpenoids, xanthones, and sesquiterpene [75,76,77]. Plant metabolites are potential therapeutic compounds, which mainly target telomerase inhibition including hTERT and hTR, telomerase substrates, and their associated proteins [78,79,80,81]. In an anti-telomerase screening study, plant secondary metabolites play a vital role in reducing telomerase activity and induce apoptosis [75,82,83]. Various in vivo and in vitro studies exhibit that secondary metabolites have a cytotoxic potential for telomerase inhibition and anti-proliferative properties. Anticancer potentials of natural products from plants on targeting telomerase are listed in Table 3.

### 3.1. Polyphenols

#### 3.1.1. Curcumin

Curcumin, one of the primary components in dried rhizome of turmeric (*Curcuma longa* L.), possesses anti-proliferating and anti-carcinogenic properties. Various studies have shown that curcumin plays a potential role in cancer prevention as well as in inducing apoptosis, and has anti-inflammatory activities through modulation of the redox status of the cell [155,156,157,158]. A study conducted by Cui et al. [159] investigated the potential role of curcumin as chemoprevention/chemotherapy for various human cancer cell lines (Bel7402, HL60, and SGC7901). They indicated that curcumin in a dose-dependent manner showed the direct inhibitory impact on cell proliferation and suppress telomerase activity in all those cancer cell lines. A similar study conducted by Chakraborty et al. [160] in leukemia cell line K-562 and Mukherjee Nee Chakraborty [102] in leukemia cell lines K-562 and HL-60 that the curcumin plays a vital role in cancer prevention and treatment by inhibiting telomerase activity, suppressing of cell viability and inducing apoptosis. In another study, Ramachandran et al. [101] also reported that curcumin can inhibit telomerase activity in michigan cancer foundation-7 (MCF-7) breast cancer cells, which may be due to down-regulation of hTERT and myelocytomatosis viral oncogene (c-myc) mRNA expression. With respect to the researchers on the effect of curcumin on nuclear localization of telomerase, Lee and Chung [161] reported that curcumin induces down-regulation of hTERT and dissociates the binding of hTERT with p23 and thereby regulates the nuclear localization of telomerase. By inhibition of nuclear translocation of hTERT during tumorigenic progression, curcumin suppresses telomerase activity. Hsin et al. [162] administered curcumin to adenocarcinomic human alveolar basal epithelial cells (A-549) and observed its anticancer activity. They emphasize that one of the mechanisms used by curcumin is its inducing of reactive oxygen species (ROS) production, resulting in inhibition of special protein 1 (Sp1) binding activity and downregulation of hTERT. Singh and Singh [163] showed that curcumin, in a dose-dependent manner, induces apoptosis and cytotoxic effects in human cervical cancer cell lines (HeLa, SiHa, CaSki, and C33A) pretreatment with estradiol. Higher doses of curcumin are administered to the cells, which counteract the proliferative response of estradiol that induces apoptosis. Based on the studies related to cancer cell lines, it can be proven that curcumin is a potential inducer of apoptosis and suppressor of telomerase activity.

#### 3.1.2. Quercetin

Quercetin is a naturally occurring polyphenol from the flavonoid groups found in most fruits (apples, grapes, berries, cherries, red wine, and citrus), vegetables (onion, tomato, sweet potato radish, capers, broccoli, and fennel), green tea, and food grains. Studies show that quercetin exhibits anti-proliferative and pro-apoptotic effects as well as anti-carcinogenic properties. Quercetin is a well-known autophagy mediator that inhibits cell proliferation by inducing cell cycle arrest, cell migration, colony formation and eventually, suppress the cancer cell progression [164,165]. Several studies demonstrate that quercetin can play an important function in cancer treatment and prevention by inhibiting telomerase activity and inducing apoptosis [166,167,168]. In colon cancer, the inhibition of growth and telomerase activity is provoked by treatment with estrogen receptor beta ligands such as quercetin and tamoxifen [169]. In a study in 2001, Choi et al. [170] stated that growth inhibition is provoked by quercetin in MCF-7 cell lines by at least two different mechanisms. Primarily, quercetin arrests the cell cycle through transient M phase accumulation followed by G2 phase arrest. Secondly, quercetin induces apoptosis. Similarly, the mechanism associated with quercetin inducing apoptosis and cytotoxic effects were observed in human promyelocytic leukemia cells (HL-60) by Kang and Seung-Eun [109] and human lung cancer cell lines by Kuo et al. [171]. They found that administration of quercetin at higher concentrations was completely arrested cell proliferation. Similarly, Lee et al. [172] administered quercetin in a dose-dependent manner to human leukemic monocyte lymphoma cells and observed increased DNA fragmentation, apoptosis and G2/M phase arrest. With respect to the research on the impact of quercetin on apoptosis, Kou [173] and Gibellini et al. [174] found that quercetin in a dose-dependent manner induces apoptosis, arrest the cells at different cycles and block their growth in various cancer cells. In addition, several animal studies have also been conducted and they found the mechanisms of chemopreventive and therapeutic effects of quercetin [175,176,177,178]. The epidemiological studies also reported that the regular consumption of quercetin (1.01–31.7 mg/day) could reduce the ovarian cancer risk [179]. Furthermore, in vivo and in vitro studies suggested that quercetin exerts anti-carcinogenic potential through inhibiting angiogenesis and tumor growth, cell cycle arrest, and inducing apoptosis [180,181]. The impact of quercetin synergizes with epigallocatechin gallate (EGCG) show anticancer potentials including death receptor 5 upregulation, activation of p53, inhibition of cell cycle, and caspase-induced apoptosis [182]. Avci et al. [167] also reported that quercetin has anti-proliferative and apoptotic effects on cells in various leukemias, such as T-cell acute lymphoblastic, acute promyelocytic, and chronic myeloid. In this study, quercetin reduces telomerase activity and apoptosis-mediated cell death and thereby it is proven as a therapeutic agent for the treatment of leukemia. Furthermore, quercetin, in a dose-dependent manner, prevents various cancer cell line growth such as lung [183], stomach [184], colon [185], nasopharyngeal [186], laryngeal [187], brain [188] and breast [189], which reduce telomerase activity, down-regulated hTERT expression and induce apoptosis. Based on various studies related cancer cell line, it can be proven that quercetin is a potential inducer of apoptosis and suppressor of telomerase activity. This result shows quercetin has a potential anti-carcinogenic effect through this mechanism.

#### 3.1.3. Resveratrol

Resveratrol (3,5,4′-trihydroxy-*trans*-stilbene) is a natural phenolic phytoalexin compound produced by various plants and in the skin of fruits, including peanut, grape, mulberry, strawberry, raspberry, and blackberry. Various studies have investigated the effect of resveratrol on telomerase inhibition activity and down-regulation of hTERT protein expression in various cancer cell lines [190,191,192]. In recent years, telomerase has become a significant therapeutic target in various cancers; inhibition of telomerase can induce senescence via the DNA damage response. Additionally, in this study, hTERT played a significant role in direct and indirect control of cell survival upon the regulation of p53 genes that function in apoptosis [192]. Treatment with Pterostilbene, as a natural analog of resveratrol, significantly decreases telomerase activity and protein expression in lung cancer cell line H460 (p53 wild-type) compared with H1299 (p53 null) cells and p53 knockdown H460 cells (H460-p53-) [192]. Another study has also shown that the effect of resveratrol on telomerase activity in human colorectal cancer cell lines [193]. Resveratrol inhibited the cell proliferation of HT-29 and WiDr cell lines and down-regulated telomerase activity in a dose-dependent manner. This study has further demonstrated that colorectal cancer has a close relationship with hTERT mRNA expression and high telomerase activity. Normally, “hTERT mRNA” is the key subunit of telomerase enzyme that is expressed in more than 85% of cancer cells, including melanoma [25], breast cancer [117,191] and adenocarcinoma [168]. In addition, resveratrol has a potential role in chemoprevention/chemotherapy for oral diseases [194], breast cancer [88] and skin carcinogenesis [195]. The chemopreventive potential of resveratrol has been attributed to a variety of mechanisms, including its general inhibition of phase I metabolism and induction of phase II metabolism [196]. Further, anticancer properties of resveratrol have shown direct inhibitory actions on the growth and proliferation of various cell, and inducing apoptosis. In this study, pterostilbene reduced the catalytic functions of telomerase, inhibited cell growth, arrested cell proliferation at S-phase and induced signaling pathways of apoptosis [192]. Studies further show that hTERT catalytic subunit can provoke telomerase activity as a result of its post-translational phosphorylation [94,125] and nuclear translocation [27,96]. Resveratrol inhibits the promoter activity of hTERT and prevented the proliferation of cells in colon cancer [197]. Zhai et al. [198] have examined the impacts of resveratrol on apoptosis, telomerase ability, and hTERT in A431 human epidermoid carcinoma cell line. In this study, resveratrol was more effective in reducing cell viability, significantly inhibited the ability of telomerase, and reduced the expression of hTERT protein in a dose-dependent manner. Pterostilbene possesses potent antitumor activity against several human cancer cell types, and is found in various plant species. Molecular docking studies have shown that pterostilbene interacts with and has high affinity for an active site of telomerase [128]. Furthermore, this study showed that the treatment of pterostilbene in MCF7 and NCI-H460 cancer cell lines exhibits significant inhibition of telomerase activity after 72 h [128]. 

#### 3.1.4. Tannic Acid

Tannic acid (TA) is a naturally occurring polyphenol found in red wine, grapes, beans, tea, coffee, nuts and various vegetables and fruits. Several studies have shown that TA has a potential activity in cancer prevention [199,200,201,202,203,204]. Cosan et al. [168] have investigated the impacts of TA on telomerase activity, cell viability, number of cells and DNA fragmentation in human breast (MCF-7) and human colon cancer (CaCo-2) cell lines. TA is effective in reducing telomerase activity, cell viability and cell count in breast and colon adenocarcinoma. Zielińska-Przyjemska et al. [199] have also reported that the anti-cancer potential of tannic acid provokes the induction of apoptosis and cell cycle in rat C6 and human T98G glioma cells. TA provokes apoptosis, which has been confirmed by phosphatidylserine externalization, cleaved caspase-3 level and loss of membrane potential in mitochondria. Other studies also found that TA arrested the cell cycle and increased the percentage of cells in the SubG1 phase in some cancer cell lines [201,205,206]. In addition, TA could protect against skin tumor promotion induced by UV radiation in an in vivo study [207]. Tietbohl et al. [208] have found that TA possesses anti-proliferative properties, which was tested in vitro against seven human cancer cells and immortalized skin keratinocytes. Animal studies have also shown that regular dietary consumption of TA has strongly demonstrated dose-dependent chemopreventive actions against hepatic tumor development and enhances the survival rate [209,210,211]. 

One of the significant TA in green tea is (−)-epigallocatechin-3-gallate (EGCG), which has been demonstrated in multiple types of cancer [212,213,214]. EGCG is a naturally occurring polyphenol from the catechin group found in tea (green, white and black), fruits (apples and plums), vegetables (onions and carobs) and nuts (hazelnuts and pecans). EGCG possibly induces apoptosis and telomerase inhibition activity, and provokes mitochondrial membrane potential and caspase-3 expression in various cancer cells [215,216,217]. In addition, EGCG has down-regulated the mRNA and protein expression of hTERT and c-Myc protein [218]. Low cytotoxic dose EGCG and (−)-epigallocatechin (EGC) have suppressed hTERT expression on reporter system and hTERT mRNA level in various cancers [213,214]. Liu et al. [217] reported that EGCG induces apoptosis by down-regulating hTERT and B cell lymphoma 2 (Bcl-2), arresting cells in both G2/M and S phase and promoting DNA damage response specifically in ovarian cancer cell lines.

### 3.2. Alkaloids

#### 3.2.1. Boldine

Boldine (1,10-dimethoxy-2,9-dihydroxy aporphine) is a natural aporphine alkaloid richly found in the boldo tree (*Peumus boldus*) and in lindera (*Lindera aggregata*). It exhibits a dose- and time-dependent cytotoxic and anti-tumor effect against various cell lines, such as liver (HepG-2), bladder (T24), and brain (U138-MG, U87-MG, and C6). The treatment with boldine in these cell lines concomitantly reduces telomerase activity, induces apoptosis and down-regulates hTERT gene expression [24,219,220,221]. A study conducted by Paydar et al. [222] in human invasive breast cancer cell lines (MDA-MB-231) and animal model shows that boldine induces cell cycle arrest at the G2/M phase and induces apoptosis, as indicated by a release of lactate dehydrogenase, membrane permeability, and DNA fragmentation. These studies promote boldine as a significant candidate for telomerase-targeted cancer and could be potent anti-cancer therapy.

#### 3.2.2. Berberine

Berberine is a benzylisoquinoline alkaloid, isolated from the roots, rhizomes, and stem bark of various plants, including *Berberis vulgaris* (barberry), *Tinospora cordifolia, Xanthorhiza simplicissima* (yellowroot), and *Coptis chinensis* (Chinese goldthread). Due to its strong yellow fluorescence, it has been decorated in the festival history of China and India, and widely used as a natural dye [223]. Previous studies conducted by Wu et al. [224] in HL-60 human leukemia cells and Naasani et al. [225] in U937 human leukemia cells show that berberine induces apoptosis with down-regulation of nucleophosmin/B23 mRNA and telomerase activity. Telomerase activity was reduced to about 35% and 63% after incubation with berberine (15 μg/mL) for 48 and 96 h, respectively. In a 2006 study, Franceschin et al. [37] stated that the inhibitory effects of berberine keeps in its preference for binding G4 with duplex DNA to become stable G4. In another study, Ji et al. [44] also reported the formation of G4 by telomeric DNA and C-Myc22 sequences, which interact with berberine and other 9 plant alkaloids. Similar studies associated with anti-telomerase effects of berberine and formation of G-quadruplex of telomeric DNA are reported by many researchers [226,227,228]. The stabilization of G4 is an important phenomenon to halt cancer cell proliferation and has been considered as a potential drug target for cancer. In this aspect, berberine is a strong affinity with G4, resulting in inhibitory effects on the telomerase activity and amplification of telo21 DNA.

### 3.3. Triterpenoid

#### 3.3.1. Pristimerin

Pristimerin is a quinone methide triterpenoid isolated from several plant species in the Celastraceae and Hippocrateaceae families that have been known to have a variety of biological activities, including chemopreventive or chemotherapeutic potentials. It has been shown to possess antiproliferative effect on various human cancer cell lines, such as breast, lung, prostate, cervical and multiple myeloma tumors [229,230,231,232,233]. Pristimerin inhibits telomerase activity and hTERT mRNA expression resulting in the suppression of native and phosphorylated hTERT protein [213]. Furthermore, the results revealed that the inhibition of hTERT mRNA expression is attributed to the inhibition of transcription factors and protein kinase that regulate hTERT post-translationally. In another study, Deeb et al. [234] also reported that pristimerin can inhibit telomerase and cell proliferative activities, arrest cells in the G1 phase and induce apoptosis in pancreatic ductal adenocarcinoma cells. Pristimerin inhibits hTERT expression by reducing the transcription factors and nuclear factor kappa beta (NF-κB), which control hTERT gene expression. Based on the data evidence, pristimerin is a potential drug candidate for various types of cancers.

#### 3.3.2. Oleanane

Oleanane (Methyl-2-cyano-3,12-dioxooleana-1,9(11)-dien-28-oate is a triterpenoid derivative of oleanolic acid with potent anti-inflammatory, anti-tumorigenic and apoptosis-inducing potential in various tumor cell lines such as breast, brain, prostate, lung, leukemia, multiple myeloma, and osteosarcoma [235,236]. Oleanane inhibits cell proliferation and telomerase suppression activity, hTERT gene expression, and a number of hTERT-regulatory protein expressions in pancreatic and prostate cancer cells [237,238]. Collectively, these results suggest that telomerase (hTERT) is a relevant target candidate of oleanane for the prevention and treatment of prostate and pancreatic cancers.

### 3.4. Xanthones

#### Gambogic Acid and Gambogenic Acid

Gambogic acid and gambogenic acid are two major secondary metabolites belonging to a family of caged xanthones which are found in gamboge resin of the *Garcinia hurburyi* tree. They have been used as coloring substances based on their unique colors. In vitro and in vivo studies have shown that gambogic acid and gambogenic acid have a broad spectrum of cytotoxic activities on numerous cancer cell lines such as prostate, leukemia, stomach, lung, breast, liver, and pancreas [239,240,241,242]. With respect to the researchers on the impact of both gambogic and gambogenic acid on apoptosis, Li et al. [243] and Fu et al. [244] reported that gambogic acid and gambogenic acid treatment significantly inhibit the proliferation of several tumor cell lines in vitro and in vivo based on doses and time. Both compounds induce apoptosis, arrest the cells at the G0/G1 phase and down-regulate the cyclin D1 and cyclooxygenase-2 expression in mRNA level. In addition, in vivo, antitelomerase activity and anticancer effects have further been shown by applying xenografts in nude mice. Several kinds of research demonstrate that gambogic acid and gambogenic acid play a significant role in prevention and treatment of cancer by suppressing telomerase activity and inducing apoptosis and thereby cell cycle arrest. Guo et al. [245] and Yu et al. [246] also reported that both compounds have potential anticancer properties, as they induce apoptosis, reduce telomerase activity and down-regulate hTERT in a post-translational manner through inhibition of the transcription activators and serine/threonine-protein kinase (Akt). Taken together, these data suggest that both compounds have antioxidant potential and may be useful, especially in combination therapies, for treating various cancers.

## 4. Conclusions

Telomerase is a diagnostic and therapeutic biomarker because it is absent from most somatic cells and is present in most cancer cells. The relationship between telomerase and cancer is complex, which makes it a distinctive target for cancer therapy. Telomerase synergistically with natural products may play a crucial role in the development of a drug for cancer therapy. Recent research clearly demonstrates that the impacts of natural compounds inhibit telomerase activity, inhibit cell proliferation, reduce hTERT mRNA and protein and subsequently promote apoptosis in various cancer cell lines. Researchers further suggest that natural products alter telomerase activity by suppression at transcriptional and post-transcriptional levels. We showed the possible relationship between natural products and working regulation of telomerase and its various binding proteins to inhibit the telomere/telomerase complex. Based on the investigation, this review concludes that natural compounds such as polyphenols, alkaloids, triterpenes, and xanthones are potential chemopreventive and chemotherapeutic agents for the treatment of cancer. 

## Figures and Tables

**Table 1:** Telomere and telomerase-associated proteins.

Protein	Functions	References
**Telomerase Components**		
Heat shock 90 kDa protein (Hsp90)	Hsp90 is a molecular chaperone, involved in the activation of disparate client proteins	[21,22,23]
Human telomerase reverse transcriptase (hTERT)	Encodes a rate-limiting catalytic subunit of telomerase that maintains genomic integrity	[24,25,26]
Human telomerase RNA component (hTERC)	Encodes the RNA component of human telomerase that acts as a template for the addition of the repeat sequence	[27,28]
Telomerase-associated protein 1 (TP1)	Associated with a catalytic subunit in a multicomponent telomerase complex	[29,30]
**Telomere Binding Proteins**		
Dyskerin,	Catalyzes pseudouridylation of rRNA required for correct intranuclear trafficking of TERC, the RNA component of the TERT enzyme	[31]
PINX1 (PIN2/TERF1-interacting telomerase inhibitor 1)	Potential telomerase inhibitor, negatively regulating telomere length by interacting with TRF1.	[32,33]
Rap 1 (Repressor activator protein 1)	Mammalian Rap1, whose function is still unclear,	[34]
TANK1 and TANK2; Tankyrase (TANK) telomere-associated poly (ADP-ribose) polymerase (PARP) 1	Positive regulator of telomere length through inhibition of TRF1	[35]
Tankyrase, TRF1-interacting ankyrin-related poly (ADP-ribose) polymerase (PARP)	Mediates poly-ADP-ribosylation of TERF1, thereby contributing to the regulation of telomere length	[36]
Telomere-end-binding protein—Protection of telomeres 1 (POT1)	Essential for the replication of chromosome termini and involved in the regulation of telomere length by cis-inhibition of telomerase	[37]
Telomeric-repeat-binding factor 1 (TRF1)	Telomere length regulation	[38]
TERF1-interacting nuclear factor 2 (TINF2)	Involves in the regulation of telomere length and protection	[39]
**Telomere Repairing Proteins**		
KU70 (Thyroid autoantigen 70 kDa (Ku antigen)	Acts as a negative regulator of telomerase and required for maintenance of the telomeric C-rich strand	[40,41]
MRE11 (Meiotic recombination 11 homologue)	A component of the MRN complex, which plays a central role in double-strand break (DSB) repair, DNA recombination, maintenance of telomere integrity and meiosis	[42]
Rad50 (*S. cerevisiae*) homologue	Single-strand endonuclease activity and double-strand-specific 3′-5′ exonuclease activity, which are provided by MRE11	[42]
Tripeptidyl-peptidase I (TPP1)	Plays a role in telomere capping by interacting with TIN2 and POT1	[43,44]
XRCC5/KU80 (X-ray repair (double-strand-break rejoining; Ku autoantigen, 80 kDa)	Works in the 3′-5′ direction and binds to DNA mediated by XRCC6	[44]
H2AX (Histone 2 AX)	Requires for checkpoint-mediated arrest of cell cycle progression in response to low doses of ionizing radiation and for efficient repair of DNA double-strand breaks	[45,46]
Ku86 (Ku autoantigen, 80 kDa)	Negative regulator of telomere length, role in telomere capping, regulation of telomerase recruitment	[47]
DNA-PK (DNA-dependent protein kinase)	Plays a role in telomere capping, putative role in post-replicative processing of telomeres	[41,48]

TERC: telomerase RNA component; TERT: telomerase reverse transcriptase; TERF1: telomeric repeat binding factor 1; TRF1: telomeric repeat factor 1; MRE11: meiotic recombination 11 homologue; TIN2: telomerase interacting nuclear factor 2; POT1: protection of telomerase protein 1; XRCC6: X-ray repair cross-complementing protein 6.

**Table 2:** Diagnostic and therapeutic implications of telomerase and telomerase inhibition on various human cancers.

Cancer	Findings	Implications	References
**Diagnostic Implications of Telomerase Activity**			
Breast	The telomerase activity in breast fine-needle aspirates has higher sensitivity (86% vs. 70% for cytology) and is detectable in stage 1 cancer cells.	Telomerase assays might play a potentially useful adjunct role in noninvasive screening and diagnosis of breast cancer.	[51]
Cervix	Telomerase activity is expressed in cervical fluid of patients.	Telomerase assay gives a promising diagnostic biomarker for early cervical cancer detection.	[54]
Colon	Telomerase is detected in the intestinal fluid of patients (80–90%) with colorectal carcinoma.	Telomerase assay holds great promising as a diagnostic biomarker for early colon cancer detection and monitoring and has considerable potential for developing anticancer therapy.	[52,59]
Kidney	Telomerase activity is expressed in kidney abscess of patients (77%) with kidney carcinoma.	Telomerase assay gives a promising diagnostic biomarker for kidney cancer detection.	[60]
Liver and biliary	Telomerase activity is expressed in liver and biliary abscess of patients (70%) with liver and biliary carcinoma.	Telomerase assay gives a promising diagnostic biomarker for early liver cancer detection.	[53,61]
Lung	The telomerase activity and circulating tumor cells in lung adenocarcinoma fluid has a higher sensitivity (78% vs. 65% for circulating tumor cells).	The combination of the circulating tumor cells and telomerase assays provide high sensitivity in lung adenocarcinoma diagnosis and follow up.	[62]
Pancreas	Telomerase activity is expressed in pancreas fluid and abscess of patients (82% and 85%) with prostate carcinoma.	Telomerase assay gives a promising diagnostic biomarker for pancreatic cancer detection.	[55,63]
Prostate	Telomerase activity is expressed in prostate abscess of patients (75%) with prostate carcinoma.	Telomerase assay gives a promising diagnostic biomarker for early prostate cancer detection.	[64]
Thyroid	Telomerase activity is expressed in thyroid abscess of patients (80%) with thyroid carcinoma.	Telomerase assay gives a promising diagnostic biomarker for early thyroid cancer detection.	[65,66]
Urinary bladder	Telomerase activity is expressed in bladder abscess of patients (80%) with bladder carcinoma.	Telomerase assays might play a potentially useful adjunct role in noninvasive screening and diagnosis of bladder cancer.	[67,68]
Uterine	Telomerase activity is expressed in uterine abscess of patients (90%) with liver and biliary carcinoma.	Telomerase assay gives a promising diagnostic biomarker for early uterine cancer detection.	[69]
**Therapeutic Implications of Telomerase Inhibition in Human Cancers by Natural Products**			
Breast	Treatment with *Melissa officinalis* extract can inhibit telomerase activity in human breast cancer cell line.	Telomerase inhibition might be useful in the treatment of various cancers with telomerase-positive cells.	[70]
Cervical	Treatment with (−)-epigallocatechin gallate can inhibit telomerase activity in human cervical cancer cell line.		[71]
Colon	Treatment with *Morus Rubra* extract can inhibit telomerase activity in human colon cancer cell line.		[72]
Liver	Treatment with *Atractylis lancea* (*Thunb*.) *DC* extract can inhibit telomerase activity in human liver cancer cell line.		[73]
Lung	Treatment with *Melissa officinalis* extract can inhibit telomerase activity in human lung adenocarcinoma cell line.		[70]
Prostate	Treatment with *Melissa officinalis* extract can inhibit telomerase activity in human prostate cancer cell line.		[70]
Uterine	Treatment with phenolic-rich extracts from *Savda Munziq* can inhibit telomerase activity in human uterine cancer cell line.		[74]

**Table 3:** Anticancer potentials of natural products from plants on targeting telomerase.

Plant Source	Compounds	Mechanism of Action	Reference
**Targeting hTERT—Inhibition of the Catalytic Function**			
*Brassica oleracea*	Indole-3-carbinol	Inhibition of telomerase and downregulated expression of the catalytic subunit of hTERT	[84]
*Camellia sinensis*	Epigallocatechin gallate	Binding competitively at the active site of hTERT	[32,33,85]
*Trigonella foenum-graecum*	Diosgenin	Prevention of telomerase activity by down regulation of the hTERT gene expression	[78,79]
*Zingiber officinale* Roscoe	Gingerol	Reduction of hTERT expression and decrease of c-Myc (myelocytomatosis viral oncogene)	[86]
**Suppression of Transcriptional and Post-Transcriptional Regulation**			
*Angelica sinensis*	Butylidenephthalide	Down-regulation of the telomerase activity and hTERT expression	[70,76,80,87,88,89,90,91,92,93,94,95,96,97]
Asian coniferous evergreen trees *Cephalotaxus* sp.	Cephalotaxus alkaloids		
*Papaveraceae*	Papaverine		
*Blueberries*	Resveratrol		
*Crocus sativus* L.	Crocin		
Marine sponge *Petrosia* sp.	Dideoxypetrosynol A		
Marine sponge *Stelletta* sp.	(Z)-Stellettic acid C		
*Melissa officinalis*	Luteolin-7-0-glucoside		
*Secondary plant metabolites*	Genistein		
Fruits and vegetables	Quercetin		
*Platycodon grandiflorum*	Saponins		
*Streptomyces* sp.	Trichostatin A		
*Streptomyces* sp.	Vinorelbine		
*Salvia miltiorrhiza*	Tanshinone I		
*Arnica montana*	Helenalin	Down-regulation of hTERT transcription through inhibition of nuclear factor kappa beta (NF-κB)	[76]
*Atractylis lancea* (Thunb.) DC.	Atractylenolide	Inhibition of hTERT expression and decreased the expression of both mRNA and protein	[73,98,99,100,101,102,103,104,105,106]
*Ganoderma tsugae*	Fungal immuno-modulatory protein-gts		
*Camellia sinensis*	Epigallocatechin gallate		
*Curcuma longa*	Curcumin		
*Laminaria japonica*	Glycoprotein LJPG (*Lamanaria japonica* glycoprotein)		
European mistletoe, *Viscum album*	Mistletoe lectin		
Cruciferous vegetables	Indole-3-carbinol		
Common fruits and vegetables	Apigenin	Inhibition of telomerase activity with down-regulation of hTERT expression, attenuating the binding of c-Myc and special protein 1 (Sp1) to the regulatory regions of hTERT	[107,108,109,110,111]
*Cordyceps militaris*	Phenolic acids		
*Dinophysis fortii*	Pectenotoxin-2		
*Garcinia hurburyi* tree	Gambogic acid	Down-regulation of hTERT transcription via inhibition of the transcription activator c-myc, and by the inhibition of the phosphorylation of serine/threonine-protein kinase (Akt); down regulation of the activity of hTERT in a post-translational manner	[112,113]
*Garlic (Allium sativum)*	Allicin and Ajoene	Reduction of hTERT mRNA levels	[114,115]
Hellbore (*Veratrum grandiflorum* O. Loes), peanuts (*Arachis hypogea*), legumes (*Cassia* sp.) and grapes (*Vitis vinifera*)	Resveratrol	Down-regulation of the telomerase activity and the nuclear levels of hTERT	[116,117]
*Vitis vinifera*	Resveratrol and pterostilbene		
*Magnolia sieboldii*	Costunolide	Inhibition of telomerase activity, reduction of hTERT mRNA and protein levels, decreasing the bindings of transcription factors in hTERT promoters	[118,119]
*Panax ginseng* C. A. MEYER, *Sun Ginseng*	Ginsenoside Rk1	Inhibition telomerase activity with down-regulation of levels of hTERT and c-Myc mRNA	[27,30,120]
*Scutellaria baicalensis*	Baicalin and wogonoside		
*Silybum marianum* L. Gaertn	Silibinin		
*Peumus boldus*	Boldine	Inhibition of hTERT expression	[24]
*Tripterygium wilfordii*	Triptolide	Inhibition of transcription of hTERT through down-regulation of transcription factor specificity protein 1	[121]
**Translocation**			
*Cottonseed*	Gossypol	Inhibition of telomerase activity with reducing the phosphorylation and nuclear translocation of hTERT	[95,96,111,122,123,124]
*Dinophysis fortii*	Pectenotoxin-2		
*Ganoderma tsugae*	Recombinant fungal immunomodulatory protein-gts		
*Secondary plant metabolites*	Genistein		
**Post-Translational Modification**			
*Broccoli* and *cauliflower*	Sulforaphane	Inhibition of telomerase activity and post-translational modification of hTERT	[122,125]
*Cottonseed*	Gossypol		
**Inhibition of Telomerase Activity**			
Red yeast rice	Rubropunctatin	Inhibition of telomerase activity	[29,77,82,83,126,127,128,129,130,131,132,133,134,135,136,137,138,139,140,141]
Mushrooms, onion, and other spices	Crude extract		
*Allium sativum* L.	Diallyl disulfide		
*Berberis vulgaris*	Berbarine		
Blueberries	Pterostilbene		
European mistletoe, *Viscum album*	Coloratum agglutinin		
*Juglans mandshurica*	Polyphenols		
Marine sponge *Dictyodendrilla verongiformis*	Dictyodendrins		
*Phyllanthus urinaria*	Gallic acid, ellagic acid, quercetin and cisplatin		
*Salvia miltiorrhiza*	Tanshinone IIA		
*Silybum marianum*	Silymarin		
*Streptomyces anulatus*	Telomestatin		
*Trichosanthes cucumerina* L.	Cucurbitacins		
Marine sponge *Axinellan fundibula*	Axinelloside A		
*Phyllanthus urinaria*	7′-Hydroxy-3′,4′,5,9,9′-pentamethoxy-3,4-methylene dioxylignan		
*Metabolites of sulforaphane from broccoli*	MTBITC(erucin)		
*Brassica oleracea*	Indole-3-carbinol and 3,3′-diindolylmethane		
*Cladonia furcate*	Lichenin CFP-2		
Diterpenoid quinone	Salvicine	Induce apoptosis and Inhibition of telomerase activity	[114,142,143]
Garlic *(Allium sativum)*	Allicin and Ajoene		
*ent-kaurene Diterpenoids*	Xerophilusin B, Macrocalin B, and Eeriocalyxin B		
*Glycine max*	Daidzein	Inhibition of cell growth and cell cycle in G2/M phase. Induce apoptosis and Inhibition of telomerase activity and reduced telomere length	[38,39,144,145]
*Panax ginseng* C.A. Meyer Radix rubra	Korean red ginseng		
*Platycodon grandiflorum*	Platycodin		
*Pedicularis striata* Pall	Verbascoside		
**Targeting hTR (human telomerase RNA component)—Transcriptional Level**			
Tabebuia avellanedae(*Lapacho tree*)	Beta-Lapachone	Inhibition of telomerase activity, down-regulation of the levels of hTR and c-myc expression	[31]
**Targeting the Telomerase Substrate and Associated Protein-Competitor for Substrate**			
*Camellia sinensis*	Epigallocatechin gallate	Binding competitively with respect to the RNA substrate primer	[32,33,85]
**G4 DNA-Interactive Compounds**			
Ascidian *Amphicarpa meridian*	Meridine	Inhibition of telomerase activity and stabilization of G4	[37,43,44,146,147,148,149,150,151,152,153]
*Berberis vulgaris chinensis (Coptis or goldenthread)*	Berberine		
*Cryptolepis triangularis*	Cryptolepine		
*Glycine max*	Daidzin		
*Glycine max*	Daidzein, daidzin, genistein and genistin		
*Menispermum dauricum and Rhizoma Menispermi*	Daurisoline, dauricinoline and daurinoline		
Okinawan tunicate *Didenum* sp.	Ascididemin		
*Boraginaceae* family (mainly in the genus of *Alkanna Lithospermum*)	Shikonin and its derivatives		
*Coptidis rhizoma*	Palmatine	Formation of C-myc22 G4 and Hum24 G4	[44,154]
*North American herb bloodroot* (*Sanguinaria canadensis*)	Sanguinarine		
